# Genome Sequences of Five African Swine Fever Virus Genotype IX Isolates from Domestic Pigs in Uganda

**DOI:** 10.1128/MRA.01018-18

**Published:** 2018-10-04

**Authors:** Charles Masembe, Vattipally B. Sreenu, Ana Da Silva Filipe, Gavin S. Wilkie, Peter Ogweng, Francis Johnson Mayega, Vincent B. Muwanika, Roman Biek, Massimo Palmarini, Andrew J. Davison

**Affiliations:** a College of Natural Sciences, Makerere University, Kampala, Uganda; b MRC-University of Glasgow Centre for Virus Research, Glasgow, United Kingdom; c College of Agricultural and Environmental Sciences, Makerere University, Kampala, Uganda; d Institute of Biodiversity, Animal Health and Comparative Medicine, University of Glasgow, Glasgow, United Kingdom; Iowa State University

## Abstract

Complete genome sequences of five African swine fever virus isolates were determined directly from clinical material obtained from domestic pigs in Uganda. Four sequences were essentially identical to each other, and all were closely related to the only known genome sequence of p72 genotype IX.

## ANNOUNCEMENT

African swine fever (ASF) is a contagious, highly lethal, hemorrhagic disease of domestic pigs caused by African swine fever virus (ASFV) (1). ASF results in up to 100% mortality. Its epidemiology is complex and adopts different patterns in Africa and Europe (2–6). The lack of effective interventions makes it extremely difficult to prevent or control and results in severe economic losses (7–11). ASFV is the sole member of the genus *Asfivirus*, family *Asfarviridae*, and has a linear, double-stranded DNA genome of 170 to 190 kbp (12, 13). There are currently 24 recognized genotypes (14, 15).

Full genome sequences enable large strides toward developing control measures to use during epidemics. Available ASFV complete genome sequences number only 20, of which 3 are from eastern Africa (Kenya) but none are from Uganda, in which ASFV is also endemic. We present the first genome sequences of strains collected from domestic pigs in Uganda.

Five domestic pig blood samples (strains N10, R7, R8, R25, and R35) were collected during a suspected ASF outbreak in 2015 from two villages in the district of Tororo, Uganda. Total genomic DNA from these samples was extracted with the DNeasy blood and tissue DNA extraction kit (Qiagen) and shown to contain ASFV sequences using a PCR assay for part of gene B646L (p72). Methylated DNA was depleted from approximately 400 ng of DNA with a NEBNext Microbiome DNA enrichment kit (New England Biolabs). The DNA was fragmented to about 450 bp with an S220 focused ultrasonicator (Covaris). Sequencing libraries were prepared with a Kapa DNA library preparation kit (Kapa Biosystems), including ligation of Illumina adapters (New England Biolabs) followed by tagging with single-index primers. The libraries were pooled in equimolar concentrations, denatured, and sequenced on a NextSeq 500 instrument (Illumina) to produce approximately 150 million paired-end reads (2 × 150 nucleotides [nt]) per sample. Trimming was done with Trim Galore 0.4.0 (http://www.bioinformatics.babraham.ac.uk/projects/trim_galore/) with a minimum Phred quality of 30 and read length of 75 nt. Reads in the quality-trimmed data were mapped to the pig genome sequence (GenBank accession number GCA_000003025.6) with SNAP aligner 1.0beta.18 (16). The remaining reads were assembled *de novo* into almost complete contigs with SPAdes 3.10.1 (17) and finished manually. Assemblies were made with Tanoti 1.3 (http://www.bioinformatics.cvr.ac.uk/tanoti.php) or Bowtie 2 2.3.1 (18), and final contig joins and corrections were made after visual inspection of the assemblies viewed with Tablet 1.14.04.10 (19). Multiple sequence alignments were generated with Decipher 2.0 (20) or GAP 4 4.11.2-r (21).

The sequences for strains N10, R7, R8, R25, and R35 had average coverages of 23, 439, 309, 869, and 1,487 reads per nucleotide, respectively, and sizes of 188,611, 188,628, 188,627, 188,630 and 188,629 bp, respectively, with an average G+C content of 38.5%. The sequences of four strains (except N10) were identical, with a few heterogeneous G · C tracts of more than 7 nucleotides for which mode sizes were incorporated. In addition to size differences in these and other repeated sequences, the sequence of strain N10 differed by 52 substitutions. A BLAST search of this data set against the NCBI nucleotide database showed the most similar GenBank sequence to be Ken06.Bus (GenBank accession number KM111295), with 99% identity and coverage of 98%. This sequence belongs to ASFV p72 genotype IX (22).

### Data availability.

The ASFV genome sequences in this communication are already publicly available in GenBank under accession numbers MH025916 to MH025920.
